# CORRIGENDUM

**DOI:** 10.1002/sct3.12983

**Published:** 2021-07-26

STEM CELLS Translational Medicine 2016;5:1644‐1655;doi: 10.5966/sctm.2015‐0373

**Corrigendum to: Induced Pluripotent Stem Cells for Disease Modeling and Evaluation of Therapeutics for Niemann‐Pick Disease Type A**

Yan Long, Miao Xu, Rong Li, Sheng Dai, Jeanette Beers, Guokai Chen, Ferri Soheilian, Ulrich Baxa, Mengqiao Wang, Juan J. Marugan, Silvia Muro, Zhiyuan Li, Roscoe Brady, Wei Zheng

The authors reported an error in the Results section of this article. In the first paragraph of the first heading, “Generation of iPSC Lines From NPA Patient Fibroblasts and NSC Differentiation”, the first sentence showed the names of the two cell lines switched. The sentence should have read as follows:

Two NPA patient fibroblast lines were reprogrammed to iPSCs, and two iPSC colonies were established for each patient line as HT138C, HT138F from **GM16195** and HT139B and HT139E from **GM13205** (Fig. 1A; supplemental online Table 1).

The two bolded cell line names were incorrect and are now corrected.

The authors regret the error and apologize for any inconvenience this may have caused.

